# Toeing the line between regeneration and fibrosis

**DOI:** 10.3389/fcell.2023.1217185

**Published:** 2023-06-01

**Authors:** Vivian Jou, Jessica A. Lehoczky

**Affiliations:** ^1^ Department of Molecular and Cellular Biology, Harvard University, Cambridge, MA, United States; ^2^ Department of Orthopedic Surgery, Brigham and Women’s Hospital, Boston, MA, United States

**Keywords:** finger, digit tip regeneration, fibrosis, blastema, limb regeneration

## Abstract

Understanding the remarkable capacity of vertebrates to naturally regenerate injured body parts has great importance for potential translation into human therapeutic applications. As compared to other vertebrates, mammals have low regenerative capacity for composite tissues like the limb. However, some primates and rodents can regenerate the distal tips of their digits following amputation, indicating that at least very distal mammalian limb tissues are competent for innate regeneration. It follows that successful digit tip regenerative outcome is highly dependent on the location of the amputation; those proximal to the position of the nail organ do not regenerate and result in fibrosis. This distal regeneration *versus* proximal fibrosis duality of the mouse digit tip serves as a powerful model to investigate the driving factors in determining each process. In this review, we present the current understanding of distal digit tip regeneration in the context of cellular heterogeneity and the potential for different cell types to function as progenitor cells, in pro-regenerative signaling, or in moderating fibrosis. We then go on to discuss these themes in the context of what is known about proximal digit fibrosis, towards generating hypotheses for these distinct healing processes in the distal and proximal mouse digit.

## 1 Introduction

Innate regeneration of lost body parts in vertebrates has long fascinated scientists in part due to the extensive need and potential for human regenerative therapies. Regenerative ability varies widely among vertebrates. For instance, axolotls are highly regenerative following amputation of their appendages and organs including the brain, spinal cord, and heart (Maden et al., 2013; Nakamura et al., 2016). Similarly, zebrafish can regenerate their fins, spinal cord, and heart (Becker et al., 1997; Poss et al., 2002). In mammals, however, regenerative ability is much more restricted. While there are examples of organ regeneration in mammals, such as liver or neonatal heart, the ability for scarless composite tissue regeneration analogous to axolotl limb or zebrafish fin is extremely limited. There are only a few examples of multi-tissue regeneration in mammals, which include deer antler regeneration, ear hole closure in rodents, and digit tip regeneration in rodents, monkeys, and humans (Waldo and Wislocki, 1951; Illingworth, 1974; Borgens, 1982; Singer et al., 1987; Gawriluk et al., 2016). The digit tip is a particularly compelling model in the context of limb regeneration, as it provides evidence that at least very distal mammalian limb tissues are competent for innate regeneration following amputation.

**FIGURE 1:**
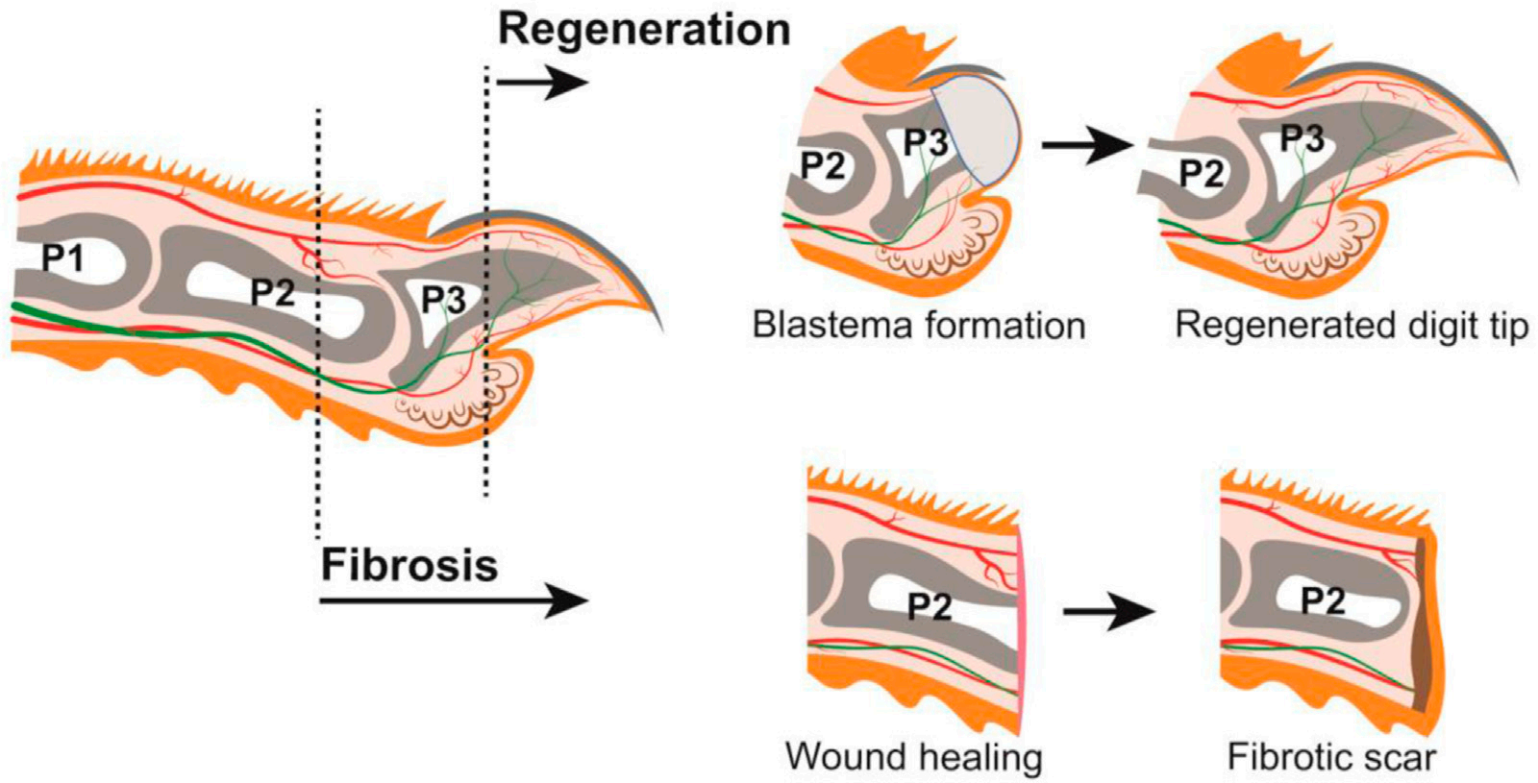
Mouse digit anatomy in relation to amputations resulting in regeneration or fibrosis (LEFT) Schematic of a longitudinal section through an adult mouse digit. Tissues depicted include: epithelium (orange), connective tissue (off-white), nail (dark grey), phalangeal bones P1, P2, and P3 (grey), bone marrow (white), sweat glands (brown), blood vessels (red), and nerves (green). Distal amputations (ex. dashed line through P3) undergo regeneration; proximal amputations (ex. dashed line through P2) undergo fibrosis. (TOP) Digit amputations that are permissive for regeneration will form a blastema (light grey) which will differentiate into the new digit tip. (BOTTOM) Digit amputations that are not permissive for regeneration will undergo wound healing and form a fibrotic scar (brown).

The regenerative capacity of the human digit tip has been recognized for nearly a century, with case reports in both children and adults (Illingworth, 1974; McKim, 1932; Douglas, 1972). This process has also been observed in rhesus monkeys and neonatal and adult rodents, such as rats and mice (Singer et al., 1987; Neufeld and Zhao, 1995; Mohammad and Neufeld, 2000). Digit anatomy and tissue composition are highly conserved among these species (Pulawska-Czub et al., 2021), and mouse has become the predominant experimental model used by researchers to study digit tip regeneration. A major component of the digit tip is the distal, third phalangeal (P3) bone which has dorsal and ventral tendons attached proximally. The P3 bone is surrounded by connective tissue populated with nerves and lymphatic and blood vessels. Epidermis encases the digit tip with specialized dorsal and ventral ectodermal appendages: the dorsal nail epithelium gives rise to the hard keratinized nail plate, and eccrine sweat glands reside in the ventral toe pad (Johnson and Lehoczky, 2021) (Figure 1). All these tissues are lost to varying degrees with digit tip amputation and are subsequently regenerated, which occurs in the following broad steps: inflammation, histolysis, wound closure, blastema formation, and differentiation (Simkin et al., 2015a). Following digit tip amputation, a blood clot forms at the wound site and inflammation occurs as immune cells, including neutrophils, natural killer cells, and macrophages, infiltrate the tissues (Simkin et al., 2017; Dastagir et al., 2022). At the later stages of the immune response, osteoclasts degrade the distal stump bone until the epidermis closes from both dorsal and ventral sides to form the wound epidermis, a structure that is a signaling source for regeneration (Lee et al., 2013; Takeo et al., 2013). Once the wound is closed, a cellular structure called the blastema forms underneath the wound epidermis, distal to the P3 bone (Figure 1). The blastema, which is the hallmark of epimorphic regeneration, is a collection of heterogeneous cells including progenitors that are the source of the regenerated tissues (Lehoczky et al., 2011; Rinkevich et al., 2011; Johnson et al., 2020; Storer et al., 2020). In adult mice, the blastema forms around 10 days post amputation (dpa), though this subtly varies by strain. Once formed, the discrete lineages within the blastema differentiate to produce a functional digit tip by 28dpa (Figure 1).

While this blastema-mediated regenerative process is robust following amputation of the distal digit tip, this is not true for the rest of the mammalian digit or limb. Successful regenerative outcome is highly dependent on the location of the amputation. Blastema formation becomes limited as the P3 digit is amputated more proximally (Dawson et al., 2021), and amputation beyond the nail matrix does not regenerate and heals via fibrosis (Neufeld and Zhao, 1993). For example, mid-digital amputation through the second phalangeal (P2) bone does not form a blastema and instead accumulates extracellular matrix leading to scarring (Agrawal et al., 2010) (Figure 1). This duality of the mammalian digit—the innately regenerative distal digit positioned immediately adjacent to the non-regenerative proximal digit—prompts several important questions. Is there a specific cell type that is required for mammalian digit tip regeneration that is missing in fibrosis? Are there defined molecular pathways or signaling factors that drive regeneration over fibrosis, or *vice versa*? And importantly, can we stimulate regeneration in innately non-regenerative tissues towards clinical applications in humans? Successful digit tip regeneration hinges on the formation of a blastema, though the pre-requisite cell types and signaling pathways are not yet fully understood. Considering the heterogeneous composition of the digit tip, individual tissues and cell-types may have discrete functions in the regenerative process. The increasing body of literature surrounding mouse digit tip regeneration supports this idea and these functions can be grouped into the following broad categories: pro-regenerative signaling, source of blastema cells, and moderation of fibrosis. In this review we discuss each of these topics in the context of distal regeneration and proximal fibrosis of the mouse digit.

## 2 Pro-regenerative signals from the nail and nerves

To understand what is different between regeneration and fibrosis, it is important to discuss the cellular signals involved in each process. Is there a cell type unique to the distal tissue that initiates or directs regeneration via external signals? Or perhaps the same cell type signals differently to the wound following distal or proximal amputations, resulting in disparate responses? To discuss the possible answers to these questions, this section describes the regenerative and fibrotic roles of the nail and nerves, the most well studied cellular signaling sources in the digit tip.

### 2.1 Cellular sources of regenerative signals in the distal digit tip

#### 2.1.1 Nail organ

Many hypotheses addressing the stark difference between distal digit regeneration and proximal fibrosis focus on the nail organ, because the presence of remnant nail in the stump tissue following amputation is one of the strongest determinants of successful digit tip regeneration (Neufeld and Zhao, 1993; Lehoczky, 2017). The nail is a distinct anatomical feature of the digit tip, covering the dorsal and lateral sides of the distal digit in mice. Nail progenitor cells have been found to reside in both the proximal nail fold and the nail matrix (Takeo et al., 2013; Leung et al., 2014; Lehoczky and Tabin, 2015). Pulse chase experiments utilizing Keratin5-TetOff driven H2B-GFP labeling identified two sources of nail progenitor cells in the homeostatic nail, slow-cycling cells in the proximal nail fold and rapidly cycling cells in the nail matrix (Leung et al., 2014). Similar experiments using Keratin14 driven LacZ expression found label-retaining nail progenitor cells in the proximal nail matrix that proliferate in the adjacent transit amplifying zone and differentiate into the nail epithelium and hard keratinized nail plate (Takeo et al., 2013). These findings were further supported by the identification of a subset of nail matrix cells expressing Lgr6, an adult stem cell marker and agonist of the WNT signaling pathway. Genetic lineage analysis showed Lgr6-expressing nail matrix cells give rise to the differentiated homeostatic nail (Lehoczky and Tabin, 2015).

**FIGURE 2:**
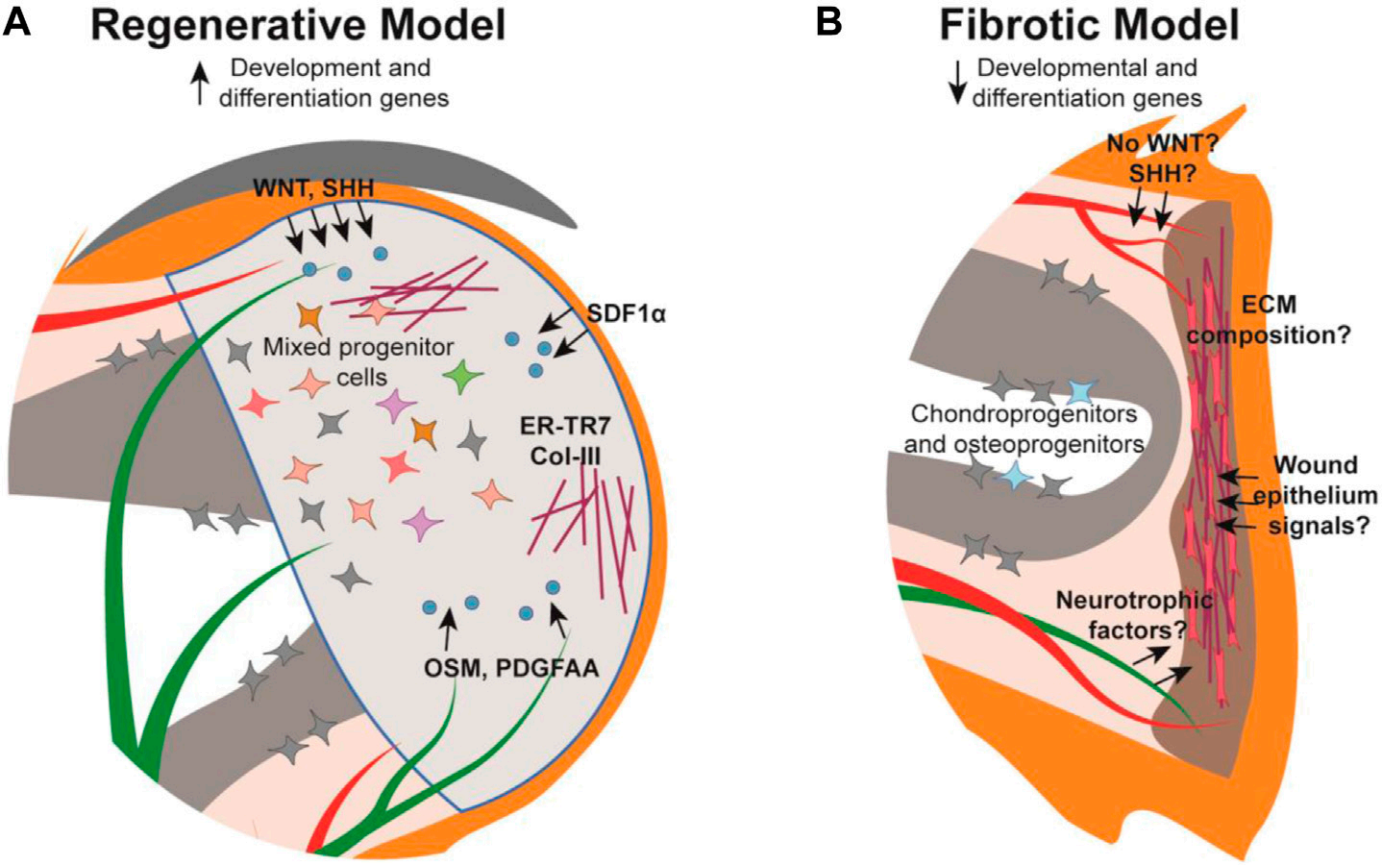
Schematic of the mouse digit tip during the regenerative and fibrotic processes. **(A)** The regenerative blastema forms distal to the amputated bone with established cellular signals (blue) from their source tissue into target tissue (arrows), extracellular matrix (maroon), and a heterogeneous mix of cells. **(B)** The digit amputated at the mid-P2 level forms a fibrotic scar distal to the wound site composed of myofibroblasts (pink) and unidirectional extracellular matrix fibers while chondro- and osteo-progenitors from the P2 bone contribute to bone repair. Cellular signalling is relatively understudied in the fibrotic model. Tissue colors are as described in Figure 1.

Following amputation of the digit tip there is a strong correlation between remnant nail matrix tissue and successful regeneration. The necessity of the nail has been demonstrated with mouse nail ablation studies whereby digit tip amputations normally permissive for regeneration, do not regenerate without the nail (Zhao and Neufeld, 1995). This finding may be partially attributed to a need for the nail stem cells, though because there is no cellular contribution of the epithelium to the digit tip blastema (Lehoczky et al., 2011; Rinkevich et al., 2011), this necessity is likely due to promoting wound epithelium formation or paracrine signaling to the underlying mesenchyme. While our understanding of the digit tip wound epithelium in this context is limited, there is evidence for the role of paracrine signaling from the nail epithelium and nail stem cells in regeneration (Takeo et al., 2013; Leung et al., 2014; Lehoczky and Tabin, 2015; Xu et al., 2021; Lao et al., 2022). Conditional deletion of β-catenin or Wntless (Wls) in Keratin14-expressing epithelia results in increased osteoclastic activity in the digit tip P3 bone, indicating that canonical WNT signaling, specifically secreted WNT ligands, originating in the epithelium functions in stimulating WNT signaling in the underlying bone during homeostasis (Takeo et al., 2016). Similarly, conditional deletion of β-catenin in Keratin14-expressing epithelial cells results in reduced nerve and osteoprogenitor recruitment within the underlying tissue, ultimately inhibiting digit tip regeneration (Takeo et al., 2013). These experiments define a broad necessity for epithelial WNT signaling in digit tip regeneration, though closer examination anchors this finding to the nail matrix (Figure 2A). Microarray analysis reveals that WNT signaling is downregulated in the proximal nail matrix, where nail stem cells are located, while WNT signaling is activated in the proliferative distal matrix (Takeo et al., 2013). Non-regenerative amputations that remove the distal matrix while preserving the nail stem cells can be rescued via stabilization of β-catenin in the epithelium. However, non-regenerative amputations that also remove the nail stem cell proximal matrix do not rescue with this same strategy, supporting the necessity of both nail stem cells and WNT signaling for digit tip regeneration (Takeo et al., 2013). These data are correlated with the finding that genetic deletion of Lgr6 results in reduced nail and digit bone regeneration (Lehoczky and Tabin, 2015).

Beyond WNT signaling, the nail epithelium has been implicated in additional signaling pathways influencing digit tip regeneration. For instance, Sonic hedgehog (Shh) was found to be expressed within the nail epithelium during regeneration leading to expression of Gli1 in the underlying mesenchyme (Maan et al., 2021) (Figure 2A). In addition, α-MSH/Melanocortin 4 receptor (Mc4r) signaling, which mediates downstream processes such as energy homeostasis, production of reactive oxygen species, and neurotrophic function, is necessary for digit tip regeneration. Mc4r is expressed in the nail matrix in the homeostatic digit, and expression expands to the blastema and regenerating nerves post amputation (Xu et al., 2021). Haploinsufficiency of Mc4r results in reduced/delayed digit tip regeneration and is severely impaired in Mc4r genetically null mice (Zhang et al., 2018; Xu et al., 2021). Collectively, the nail-focused experiments detailed to this point implicate the nail matrix as the pro-regenerative tissue for regeneration, though a recent study presents a role for the distal nail bed epithelium as well (Lao et al., 2022). Sox9 is expressed in the distal nail epithelium and when conditionally genetically deleted during homeostasis, results in hyperproliferation of the nail matrix and extremely long nail plates. When Sox9 is conditionally deleted during digit tip regeneration, the bone fails to regenerate, though it remains to be determined whether this is due to the influence of the nail or a Sox9-expressing mesenchymal population (Lao et al., 2022).

#### 2.1.2 Distal digit nerves

Several of the studies detailed above reveal a role for the nail epithelium in maintaining and regenerating the underlying mesenchyme, specifically the bone (Takeo et al., 2013; Takeo et al., 2016; Lao et al., 2022). Separately, much of the data support a pro-regenerative function for the nail epithelium in recruiting nerves to the regenerating digit tip (Takeo et al., 2013; Xu et al., 2021). For example, the digits that failed to regenerate following conditional deletion of β-catenin or Wls in epithelial cells had fewer nerves recruited to the wound site mesenchyme compared to controls, consistent with reduced Sema5a expression in the nail epithelium (Takeo et al., 2013). Indeed, nerves are critical for regeneration in many vertebrate limb and appendage models, including axolotls and zebrafish (Kumar et al., 2007; Simões et al., 2014). Denervation in these models results in the lack of a blastema and ultimately failed regeneration. Through mechanistic studies, it was established that in these species, nerves secrete factors such as Transferrin and Neuregulin-1 to promote regeneration (Mescher et al., 1997; Farkas et al., 2016). Investigation into the neuroanatomy of the mouse digit tip via immunohistochemistry reveals a complex network of sensory and motor nerves that are regenerated following amputation, though in fewer numbers and with different patterning than the unamputated digit tip (Dolan et al., 2019). In rat, denervation via transection of the sciatic nerve delays digit tip regeneration, whereby the denervated digits had significantly less bone and nail length than controls at 13dpa, but were comparable by 28dpa (Mohammad and Neufeld, 2000). Delayed regeneration is also found with mice; digit tip regeneration following denervation was delayed at 28dpa, but had comparable bone volume to controls at 42dpa (Dolan et al., 2022a). These experiments suggest transient nerve dependence for blastema formation or proliferation, but other studies point to the necessity of innervation for the entirety of digit tip regeneration. These experiments show that denervated mice had significantly less nail and bone *versus* control mice at the completion of digit regeneration (Takeo et al., 2013; Johnston et al., 2016), while another study revealed denervation led to malformed bone and nail in the regenerated digits (Rinkevich et al., 2014). The variation in regenerative outcomes in these studies could be due to individual denervation techniques and timing in relation to amputation, but collectively these data support a pro-regenerative function for nerves in the digit tip during regeneration (Mohammad and Neufeld, 2000; Rinkevich et al., 2011; Takeo et al., 2013; Johnston et al., 2016; Dolan et al., 2022a).

Much remains to be understood regarding how nerves promote digit tip regeneration, though it has been determined that at least part of their necessity lies with the nerve-associated Schwann cell precursors (SCPs). Sox2-expressing SCPs populate the digit tip blastema prior to regenerating axons, but in denervated limbs these SCPs were not found in the blastema and digit tip regeneration was inhibited (Johnston et al., 2016). Follow-up experiments determined that conditional genetic deletion of Sox2 or conditional DTA-mediated ablation of Sox2-expressing cells during digit tip regeneration both resulted in regenerative failure, demonstrating the phenotype is specific to Sox2-expressing SCPs. RNA analysis and cell-surface proteomics from cultured rat sciatic nerve SCPs identified OSM and PDGF-AA as pro-regenerative growth factors (Johnston et al., 2016) (Figure 2A). However, neurotrophic factors from the peripheral nerves themselves have yet to be identified in the digit tip and may be key in regeneration as they are in other systems.

### 2.2 Nails and nerves in the context of the proximal digit

**FIGURE 3:**
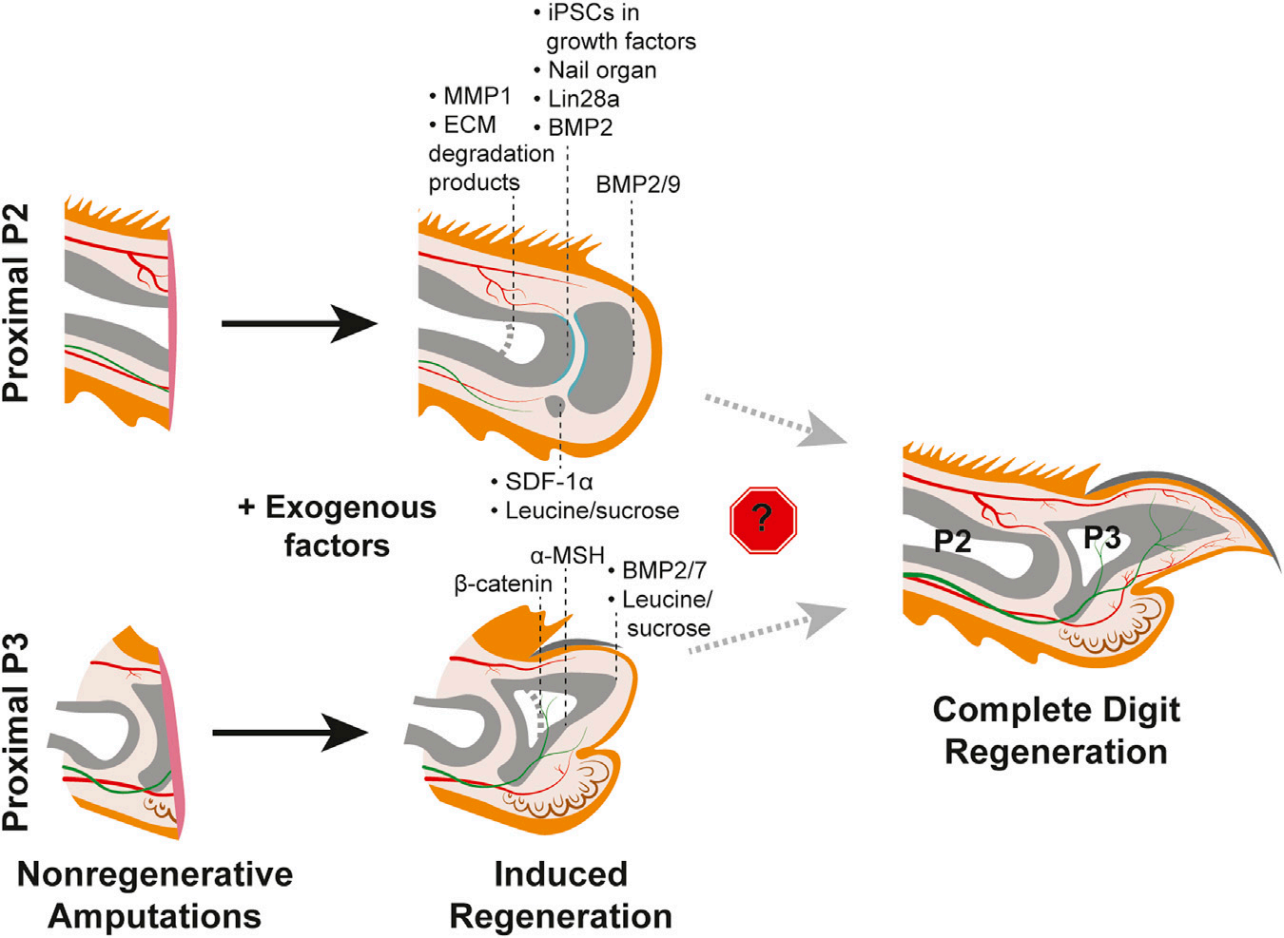
Efforts to induce regeneration in nonregenerative mouse digit amputation models. Studies utilizing the P2 amputation model (TOP) and nonregenerative proximal P3 model (BOTTOM) with post-amputation treatment with exogenous factors yield varying degrees of regenerative success as indicated by the black dashed lines for each factor. Induced regeneration of the chondrogenic joint is shown in teal. Thicker light grey dashed lines within the phalangeal bones indicate original amputation plane. No study has yet to identify factors that overcome the roadblock(s) to regenerating the full mouse digit tip, as shown on the right. Tissue colors are as described in Figure 1.

Why would distal, but not proximal, digit amputations innately regenerate? In the context of pro-regenerative signals originating from the nail organ or nerves, perhaps there are specific signaling pathway(s) or cell-type(s) that are strictly required for regeneration but absent in the proximal digit? The absence of the nail organ in the proximal digit could fit this hypothesis and potentially explain the different regenerative outcomes (Lehoczky, 2017). Intriguingly, grafting of a nail onto a P2 amputated digit in juvenile rats resulted in increased bone regeneration, supporting the pro-regenerative influence of the nail organ (Mohammad et al., 1999) (Figure 3, Table 1). However, the nail graft-induced regenerated bones in these experiments did not recapitulate the pre-amputation proximal bone morphology, indicating that the nail organ alone is not sufficient to induce complete digit regeneration. As detailed above, much of the pro-regenerative effect of the nail in distal digit tip regeneration can be attributed to WNT signaling, which would suggest that with the absence of the nail on the proximal digit there is low or no innate WNT signaling following amputation. While a comprehensive analysis of WNT expression in P2 digits during homeostasis or fibrosis has not been reported, it has been found that unlike in distal regenerative P3 amputations, epithelial WNT signaling is not activated following a non-regenerative proximal P3 amputation removing the distal nail matrix (Takeo et al., 2013) (Figure 2B). Ectopic epithelial expression of β-catenin in mice with similar non-regenerative P3 amputations was sufficient to stimulate regeneration and recruit more nerves and proliferating cells as compared to controls (Takeo et al., 2013). However, these regenerated digits did not fully return to the pre-amputation nail and bone lengths, and K14-creER; Bcat-fl/ex3 induction of WNT signaling did not stimulate regeneration when the amputations also removed the proximal nail matrix (Figure 3; Table 1). Together, these experiments indicate that WNT signaling alone is not enough to fully induce regeneration (Takeo et al., 2013).

**TABLE 1:** Exogenous factors tested for sufficiency to induce regeneration in proximal, non-regenerative digit amputations.

Exogenous factor	Amputation level	Age of rodent	Delivery method	Results	References(s)
Nail organ	P2	7–21-day old rat pups	Transplantation	Increased bone growth and formation of nail-like structure	Mohammad et al. (1999)
iPSCs with growth factors (BMP2, FGF8, Tβ4, Wnt3a)	P2	8–10-week-old adults	Transplantation	Longer P2 bone regenerated	Chen et al. (2017)
MMP1	P2	5-week-old adults	IP injection	Improvement in soft tissue regeneration	Mu et al. (2013)
BMP2	P2	PN3 neonates and 8-week old adults	Agarose beads	Regeneration of the P2 bone	Yu et al. (2012), Dawson et al. (2017)
BMP2/9	P2	PN3 neonates and 4–8-week-old adults	Agarose beads (sequential treatment)	Regeneration of P2/P3 joint with a distal bone nodule	Yu et al. (2019)
ECM degradation products	P2	6–8-week-old adults	Local injection	Increased in progenitor cells, decreased in collagen, ectopic bone nodule formation	Agrawal et al. (2011a), Agrawal et al. (2011b), Agrawal et al. (2012)
SDF-1α	P2	PN3 neonates	Transplantation of SDF-1α expressing cells	Longer P2 bone regenerated, ectopic sesamoid bone formation	Lee et al. (2013)
Lin28a	Proximal P3	PN2 neonates	Conditional genetics	Accelerated digit regrowth in soft tissue and bone	Shyh-Chang et al. (2013)
BMP2/7	Proximal P3	PN3 neonates	Agarose beads	Improved bone regeneration	Yu et al. (2010)
α-MSH	Proximal P3	PN3 neonates	IP injection	Improved bone regeneration	Xu et al. (2021)
β-catenin	Proximal P3	3-week-old adults	Conditional genetics	No improvement in digit regeneration	Takeo et al. (2013)
Leucine/sucrose	P2 and Proximal P3	3-6-week-old adults	Water supplement	Increased bone regeneration and ectopic bone regrowth	Abrams et al. (2021)

A separate study found that Melanocortin 4 receptor (Mc4r), an important regulator of energy homeostasis, is expressed in the nail matrix and underlying mesenchyme during innate regeneration in the distal digit. It was previously shown by genetic deletion that Mc4r is critical for digit tip regeneration (Zhang et al., 2018). These studies suggest that lack of regeneration in the proximal digit could be due to the lack of Mc4r expression or a failure to modulate energy balance following amputation. Consistent with this hypothesis, injection of α-MSH, an agonist of the Mc4r signaling pathway, was found to increase tissue regeneration in proximal, non-regenerative P3 amputations (Xu et al., 2021) (Figure 3, Table 1). However, as was found with proximal over-expression of WNT signaling (Takeo et al., 2013), α-MSH induced regenerates did not regain the total length or morphology of innate regeneration controls (Xu et al., 2021), indicating that the Mc4r signaling pathway is not solely sufficient to induce regeneration. It follows that Mc4r signaling may fit into a broader need for increased energetics to stimulate regeneration; oxaloacetic acid treatment accelerates regeneration in distal digit amputations, conditional overexpression of Lin28a increases proximally amputated digit regrowth rate, and leucine and sucrose treatment following P2 amputation increases regeneration (Shyh-Chang et al., 2013; Abrams et al., 2021; Tower et al., 2022; Jaramillo et al., 2023) (Figure 3, Table 1). However, whether the overall metabolic profile of a distal amputation is unique to regeneration and if specific metabolic factors are a driving factor for regeneration are still unknown.

Unlike the anatomical restriction of the nail organ, the entire digit is innervated, thus it is unclear why nerves would respond differently following distal and proximal digit amputations (Figure 1). Importantly, immunohistochemical analyses of the distal digit tip revealed a reduction in connective tissue branch nerves and associated myelinating Schwann cells following regeneration, demonstrating that normal nerve regeneration in the digit tip is imperfect (Dolan et al., 2019). A complementary analysis has not been reported for the proximal digit, but understanding the neuroanatomy of the proximal region in homeostasis and fibrosis could provide important insights as to how nerves respond differently in the two regions, whether it is sensitivity to pre-existing anatomy, local environmental cues, or something else entirely. scRNAseq of the non-regenerative proximal digit offers information into the nerve-associated cell content of the fibrotic environment (Storer et al., 2020). These data identify Schwann cells, a cell-type necessary for successful digit tip regeneration, present in the proximal fibrotic tissue (Johnston et al., 2016; Storer et al., 2020). By bulk RNAseq analysis, these proximal post-amputation Schwann cells are not significantly different than Schwann cells in the distal blastema, though pro-regenerative Schwann cell secreted factors such as OSM and PDGF-AA have not yet been addressed in the context of proximal fibrosis (Johnston et al., 2016; Storer et al., 2020) (Figure 2B).

## 3 Connective tissue and extracellular matrix during regeneration

### 3.1 The necessity and contribution of connective tissue to distal digit regeneration

It is well-established that the blastema is a critical component of epimorphic regeneration. Studies in non-mammalian epimorphic limb regeneration models, such as the axolotl, demonstrate that limb connective tissue is the main contributor of both cells and patterning information to the blastema (Stocum, 1982; Muneoka et al., 1986a; Muneoka et al., 1986b; Lin et al., 2021). In the context of the mouse digit tip, the term “connective tissue” broadly encompasses heterogeneous fibroblasts [including mesenchymal progenitors, reticular fibroblasts, nail bed mesenchyme, and nerve-associated mesenchyme (Wu et al., 2013; Marrero et al., 2017; Carr et al., 2019; Johnson et al., 2020; Mahmud et al., 2022)], and skeletal cells [including tendon-associated cells, periosteal cells, and bone-associated cells (Lee et al., 2013; Dawson et al., 2016; Johnson et al., 2020)]. Failure of digit tip regeneration following genetic deletion of Msx1 or conditional ablation of Pdgfrα-expressing cells supports the importance of connective tissue for this process (Han et al., 2003; Storer et al., 2020). However, given the diversity of cell-types defined by these genes, studies focused on specific connective tissue subpopulations can offer additional granularity into the function of connective tissue in digit tip regeneration.

#### 3.1.1 Distal digit fibroblasts

Fibroblasts make up the majority of cells in the digit tip blastema (Johnson et al., 2020). Both immunohistochemistry and single cell RNA sequencing-based analyses demonstrate extensive heterogeneity among this population, suggestive of diversity in function during regeneration (Marrero et al., 2017; Carr et al., 2019; Johnson et al., 2020; Storer et al., 2020; Mahmud et al., 2022). One of these functions is to serve as a cellular source, i.e., progenitor cells, for regenerating connective tissues. This has been demonstrated with genetic lineage analyses of Prx1-, Msx1-, or Pdgfrα-expressing cells which collectively reveal that digit tip fibroblasts are not pluripotent progenitors for the regenerating digit tip, but instead progenitors that remain fate restricted to cell types of mesenchymal origin (Lehoczky et al., 2011; Rinkevich et al., 2011; Storer et al., 2020). These experiments not only show that digit tip fibroblasts give rise to the majority of cells in the blastema (Lehoczky et al., 2011; Storer et al., 2020), but also that Pdgfrα-expressing cell descendants acquire a unique mesenchymal progenitor state in the blastema, as determined by scRNAseq (Storer et al., 2020). However, it remains to be determined how this newly defined progenitor state is established or if this transcriptional reprogramming is required for digit regeneration, as found for axolotl limb regeneration (Lin et al., 2021). A separate study used scRNAseq fibroblast subpopulation size dynamics throughout digit tip regeneration to determine if all subpopulations regenerate on the same time scale (Johnson et al., 2020). These analyses define four blastema-enriched fibroblast subpopulations and associated differentially expressed genes, supporting the hypothesis that a subset of digit tip fibroblast subtypes may be pro-regenerative (Johnson et al., 2020).

Another major role of fibroblasts is to deposit extracellular matrix (ECM), a crucial non-cellular component of the connective tissue. It follows that the difference between the organized ECM of scar-free regenerated digit tips and the dense, disorganized ECM deposited during scarring in non-regenerative digit amputations, may be attributable to different fibroblast responses (Figure 2). Indeed, experiments with *in vitro* cultured proximal (P2) and distal (P3) derived fibroblasts revealed that P3 fibroblasts were unable to contract, which is a key mechanistic response during fibrosis, but it was observed with the P2 fibroblasts (Wu et al., 2013). This is compelling evidence that differences in ECM composition, or the type of fibroblasts that produce it, are key factors in regenerative response. However, this study was performed prior to the rise of single-cell technology, which defined extensive heterogeneity in fibroblasts (Johnson et al., 2020; Storer et al., 2020). Thus, whether the difference in contractile ability is consistent among all fibroblast subtypes or only specific to certain ones is unclear. This role for ECM in regeneration *versus* fibrosis is further supported by the identification of reticular fibroblasts and associated ER-TR7-positive ECM in the digit tip and blastema (Marrero et al., 2017). The increase in ER-TR7 expression was also correlated with Collagen-3, an ECM component that is typically associated with scar-free healing in fetal wounds and a regenerative response in the spiny mouse (Leung et al., 2012; Seifert et al., 2012; Marrero et al., 2017) (Figure 2A).

Fibroblasts and the associated ECM can also function to retain positional identity in the limb. In salamanders, fibroblasts and the ECM have been found to harbor the positional information necessary for intercalation and proper patterning of the regenerating limb, though how fibroblasts encode this information remains an active area of investigation (Bryant et al., 1987). There are hints that mouse limb ECM functions by a conserved mechanism in that a heparan sulfate dependent pathway was found to distinguish anterior and posterior fates when grafted onto axolotl blastemas (Phan et al., 2015). Additionally, the endogenous expression of heparan sulfate sulfotransferases correlated with higher heparan sulfate activity in the anterior region of developing mouse limbs (Phan et al., 2015). However, the ability of mouse ECM to establish positional identity during digit tip regeneration remains to be explored.

Beyond ECM production, fibroblasts can participate in patterning during regeneration via induced gene expression networks. For example, following axolotl limb amputation, fibroblasts dedifferentiate to an embryonic limb-bud-like state and re-initiate the limb development patterning program in the blastema (Lin et al., 2021). In mice, the digit tip similarly regenerates with proper morphology, though there is subtle variation in bone length and nerve organization/number (Fernando et al., 2011; Dolan et al., 2019). It has yet to be determined how this re-patterning occurs, though recent studies suggest that these species may utilize different patterning mechanisms. Mouse scRNAseq analyses of the digit tip blastema compared to embryonic digit and postnatal digit tip cells found that while mesenchymal cells do undergo a transcriptomic change, this is a unique regenerative state and is different from that of embryonic or developing digit cells (Storer et al., 2020). This suggests that, unlike the axolotl, mouse digit tip regeneration does not utilize the same processes as in development. This conclusion is further supported by the finding that embryonic dorsal-ventral limb patterning genes, En1 and Lmx1b, are not necessary for dorsal-ventral patterning during digit tip regeneration, as demonstrated by conditional genetic knock-out during regeneration (Johnson et al., 2022). However, in the absence of developmental patterning gene network reactivation, it is yet to be determined how dorsal ventral patterning of the connective tissue is re-established during digit tip regeneration.

#### 3.1.2 Distal digit tip bone

The distal phalangeal (P3) bone is the major internal component of the digit tip. An amputation permissive for regeneration can remove over half of this bone, thus a significant amount of regeneration is required (Figure 1). Following amputation, osteoclasts degrade the stump bone, ultimately exposing the marrow cavity to the wound environment (Fernando et al., 2011). Termination of P3 bone histolysis is regulated by epidermal closure, as was demonstrated by induced attenuation following premature wound closure with Dermabond (Simkin et al., 2015b). Aging can also modulate the duration of this phase of enhanced osteoclastic activity. One year old mice have significantly delayed wound closure, resulting in prolonged histolysis and lower regenerate bone volume than young mice controls (Brunauer et al., 2021). It is not clear what role histolysis has in digit tip regeneration, as it seems counterintuitive to remove additional bone prior to regenerating it. Several studies point to the bone marrow stroma as a cellular contributor and source of molecular signaling to the blastema (Fernando et al., 2011; Lee et al., 2013; Dawson et al., 2018), supporting a pro-regenerative role for P3 bone histolysis following distal digit amputation.

The blastema forms following histolysis and epithelial closure, and among other progenitor cell types, it contains a population of osteoprogenitors (Han et al., 2008; Lehoczky and Tabin, 2015; Johnson et al., 2020) (Figure 2A). Because the P3 bone regeneration encompasses a large part of digit tip regeneration, defining the source(s) of blastemal osteoprogenitors is important for understanding how the distal digit is competent for regeneration. Significant progress has been made on this front. Histology and immunohistochemistry based experiments support blastemal osteoprogenitors originating from the bone marrow stroma and endosteum (Simkin et al., 2015b; Dawson et al., 2018) (Figure 2A). However, additional source(s) are likely because premature wound closure, which attenuates histolysis and opening of the bone marrow cavity, still results in bone regeneration (Simkin et al., 2015b). Consistent with this, the P3 periosteum contains Runx2-expressing, proliferative cells post-amputation, and was found to be required for regeneration as determined by surgical removal resulting in significantly less bone regeneration (Dawson et al., 2018). These studies are complemented by several independent genetic lineage analyses for the digit tip bone. Sp7-expressing pre-amputation lineage-marked osteoblasts give rise to the regenerated P3 regenerated bone and periosteum, supporting the endosteum and periosteum as the source of osteoprogenitors (Lehoczky et al., 2011). Separately, Sox9 embryonic lineage-marked bone, as well as Dmp1-expressing pre-amputation lineage-marked bone cells, were found to contribute to the P3 regenerate (Rinkevich et al., 2011; Storer et al., 2020). These data support not only the endo/periosteum as a source of osteoprogenitors, but potentially also osteocytes within the digit tip bone. Interestingly, the Dmp1 lineage marked cells were also found to contribute to the regenerated digit tip dermis which hints at the idea of differential plasticity among osteoprogenitors in the digit tip (Storer et al., 2020).

While the origin and plasticity of digit tip blastema osteoprogenitors continues to be refined, it has been determined that they differentiate into the new digit tip bone via intramembranous ossification, which is a distinct mechanism from the endochondral ossification by which the digit tip develops (Han et al., 2008; Fernando et al., 2011). Digit tip bone regeneration is considered imperfect due to the production of trabecular bone with more volume and length than the original (Fernando et al., 2011). However, the regenerated bone has proper dorsal-ventral asymmetry, indicating the process is not simply stochastic bone repair (Johnson et al., 2022). While digit tip bone regeneration is a robust and faithful process, there are several factors that can attenuate the response. For instance, aging slows the ability of the blastema to differentiate into bone such that old mice require an additional month of regeneration as compared to young mice controls (Brunauer et al., 2021). It was also shown that repeated amputation of the same digit tip reduced the number of osteoblasts and proliferative index of the blastema, which ultimately led to less bone regeneration (Dolan et al., 2022b). This finding suggests that multiple amputations negatively impact the overall ability of the blastema to differentiate into bone. Taken together, these studies underscore the importance of a sufficient osteoprogenitor population and proliferative rate for successful innate regeneration of the digit tip.

### 3.2 Connective tissue, ECM, and the bone in the context of the proximal digit

When attempting to understand why the proximal digit undergoes fibrosis instead of regeneration following amputation, it is important to consider if the connective tissue mediates these different responses. Why do fibroblasts undergo a contractile response in the proximal digit and not the distal (Wu et al., 2013)? Perhaps they have distinct innate regenerative potential between the two regions. Or maybe all fibroblasts are capable of regeneration, but it is a lack of pro-regenerative signals that leads to fibroblast contraction and fibrosis. The bone could also be a driving factor between the differential responses, particularly as a source of progenitor cells. This section details the current progress in understanding the limitations of proximal digit regeneration in the context of mouse digit tip connective tissues.

#### 3.2.1 Proximal digit fibroblasts

Distal digit fibroblasts contribute extensively to the regenerative blastema, but no blastema forms following proximal digit amputation and the subsequent wound-healing ends in a fibrotic scar. This suggests that fibroblasts could have a critical role in the decision between regeneration and fibrosis. While this disparate fibroblast response remains an open question, studies have begun to shed light on potential differences. Bulk RNA sequencing found an upregulation in ECM-related genes and myofibroblast differentiation genes in proximally amputated digits when compared to distal regenerating digits (Maan et al., 2021). Furthermore, single cell RNA sequencing analyses of P2 nonregenerative connective tissue found that the nonregenerating cells are transcriptionally distinct from distal regenerative mesenchymal cells at both 10dpa and 14dpa (Storer et al., 2020) (Figure 2). While this suggests that proximal and distal fibroblasts have opposite regenerative capacities, deeper analysis indicates proximal fibroblasts undergo transcriptomic changes toward a slight regenerative state. Intriguingly, these cells expressed 44% of the newly-defined blastema signature genes, and pseudotime trajectory analysis placed the P2 cells in between the uninjured and regenerative P3 cells (Storer et al., 2020). Whether these findings suggest that these genes are shared between fibrosis and regeneration or if the proximal fibroblasts are missing crucial signals to fully commit to regeneration requires further investigation.

The distinct transcriptome profiles of proximal P2 and distal P3 fibroblasts following amputation indicate that they could have intrinsic regenerative differences based on digit position. This is also supported by the differing regenerative phenotypes of *ex-vivo* cultured P2 and P3 fibroblasts (Wu et al., 2013). P2-derived fibroblasts exhibited high contractility when seeded in collagen gels, a phenotype associated with fibrosis, which was contrasted by the low contractility found with P3-derived fibroblasts (Wu et al., 2013). This suggests these innate, location-of-origin-based behaviors may underlie the larger decision between digit regeneration and fibrosis. Indeed, when P2-derived fibroblasts were injected into distal regenerating digit tips, they had lower proliferation indices and higher apoptosis rates as compared to endogenous P3 fibroblasts. However, P3-derived fibroblasts were not sufficient to rescue fibrosis when injected into proximally amputated digits, supporting the need for additional factors in shifting fibrosis towards regeneration (Wu et al., 2013).

Beyond position-dependent cell intrinsic differences in digit fibroblasts, the wound environment is a likely factor in determining fibrosis or regeneration. The finding that proximally-derived cultured fibroblasts were competent to engraft in the distal digit tip and participate in blastema formation, despite distinct P2 and P3 fibroblast phenotypes, points to a pro-regenerative environment in the distal digit (Wu et al., 2013). Similarly, fibroblasts isolated from mouse skin transplanted into post-amputation digits, were found to have extremely different gene expression profiles when in regenerating or non-regenerating wounds (Storer et al., 2020). While those in the proximal fibrotic environment did not express any blastema signature genes, those in the distal regenerative environment did, and went on to cellularly contribute to the blastema and regenerating connective tissues (Storer et al., 2020). Indeed, there is evidence that the wound environment functions in determining tissue composition and cell fate. For example, mice with proximal amputations treated with MMP1, a modulator of the ECM shown to degrade collagen at wound sites, exhibited improved soft tissue regeneration. These digits were found to have an increased number of Sca1+ progenitor cells and neuromuscular junctions along with decreased collagen deposition (Mu et al., 2013) (Figure 3, Table 1). Separately, it was found that proximal digit amputations treated with ECM degradation products in the form of cryptic peptides or pepsin digest cocktails improved tissue regeneration (Agrawal et al., 2012). These digits had an increase in cellular density, and those expressing mesenchymal stem cell markers Sox2, Sca1, and Cd90, had higher amounts of proliferative cells and exhibited increased bone formation (Hechavarria et al., 2010; Agrawal et al., 2011a; Agrawal et al., 2011b; Agrawal et al., 2012) (Figure 3, Table 1). These studies indicate that the extracellular matrix may directly control the fate of the tissue either towards fibrosis or towards regeneration. However, the specific differences between the proximal and distal extracellular matrix composition and overall wound environment still require much investigation.

#### 3.2.2 Proximal digit bone

Unlike the regenerating distal digit, the P2 bone does not reform the missing distal portions of the bone in a proximal amputation. While the bone does not recover any length along the proximo-distal axis, there is new bone deposition along the circumference of the injured bone, resulting in increased bone volume. Chondroprogenitors from the periosteum form a chondrogenic callus, which mediates this circumferential expansion of the bone. Similar to regeneration, both the endosteum and periosteum also contribute osteoprogenitors for bone formation during fibrosis (Figure 2B). The P2 amputation and bone fracture responses were found to be extremely similar, with the exception that there is no skeletal elongation following amputation. These results provide a foundation for understanding the different bone responses in fibrosis and regeneration (Dawson et al., 2016).

As the major internal component of the digit tip, most studies that aim to stimulate proximal regeneration focus on stimulating bone reformation, which will then support the regeneration of the other tissues. One such attempt inserted gelatin containing BMP7 to proximally amputated limbs, which resulted in the formation of additional bone elements at the wound site (Masaki and Ide, 2007). This outcome was corroborated by a separate study, in which BMP-soaked beads were utilized to deliver BMP2 and BMP7 to proximal digit amputations, resulting in modest bone regeneration (Yu et al., 2010) (Figure 3, Table 1). Separately, BMP7 was reported to induce some bone regeneration following mouse limb amputation while BMP2 induced P2 bone regeneration in both neonates and adult mice via formation of a distal ossification center (Ide, 2012; Yu et al., 2012; Dawson et al., 2017) (Figure 3, Table 1). A challenge with the proximal digit model is to regenerate the joint between the P2 and P3 bone, which has recently been addressed (Yu et al., 2019). P2 amputated digits treated with BMP9 soaked beads exhibited chondrogenesis as well as joint cavitation, which was not found in controls. Intriguingly, treatment with BMP2 regenerated the P2 bone and sequential treatment with BMP9 induced chondrogenic differentiation to form the joint, which highlights how regeneration is an intricate, multi-step process (Yu et al., 2019) (Figure 3, Table 1). While these experiments support the sufficiency of exogenous BMPs to induce proximal bone regeneration, additional factors and/or cell sources may be necessary for regeneration of other cell types. To this point, it was found that transplantation of iPSC-derived limb progenitor-like cells with exogenous factors including BMP2, FGF8, Tβ4, and WNT3a increased proximal digit bone regeneration and the transplanted cells contributed to bone as well as soft tissue (Chen et al., 2017) (Figure 3, Table 1). Treatment of proximal amputations with the growth factors alone only minimally induced regeneration, suggesting that perhaps there is a specific lack of progenitor cells in the P2 tissue that are present in the distal region (Chen et al., 2017). Collectively these studies show that while the proximal amputated bone undergoes repair similar to bone fracture, the failure to elongate may be due to a lack of regenerative signals directing the growth as well as a lack of progenitors in the tissue.

## 4 Discussion

One of the greatest benefits of the mouse digit tip as a regenerative model is that there is a nonregenerative model directly adjacent to the regenerative one (Figure 1). Amputations proximal to the nail matrix result in failed regeneration, thus the digit allows for direct comparison of two drastically different outcomes in a close anatomical space.

### 4.1 Gaps in our knowledge

There are several major cell types that have been studied in the context of distal digit tip regeneration that have not yet been explored in the context of proximal digit fibrosis. For example, immune cells which infiltrate the digit tip immediately after amputation to fend off foreign bodies and infection. This immune response involves a variety of immune cells which provide growth factors and cytokine signals to promote tissue repair. The digit tip inflammatory response follows a similar cellular response to canonical wound healing, in which neutrophils are first to enter the wound site and are followed by macrophages and natural killer cells. Mice that had their macrophages depleted exhibited abnormal or failed digit tip regeneration due to an inhibition of bone histolysis and wound closure (Simkin et al., 2017). Similarly, natural killer cells are also necessary for digit tip regeneration, as ablation leads to delayed bone histolysis and delayed bone formation (Dastagir et al., 2022). There are still major areas of uncertainty pertaining to the inflammatory response in proximal digit amputations. What does the inflammatory response look like in the fibrotic wound healing process compared to the digit tip? Are there any unique immune cell populations or cytokine signals in either the P2 or P3 tissues driving fibrosis *versus* regeneration? A comprehensive understanding of these questions may help illuminate the initial decision-making process of regeneration *versus* fibrosis.

Another major cell type understudied in the proximal fibrotic response is wound epithelial cells. The wound epithelium in other regenerative species is considered a transient, pro-regenerative tissue, distinct from homeostatic or nail epithelia. In digit tip regeneration, the wound epidermis closes over the amputation wound, ending bone histolysis, transitioning the digit to blastema formation. In the regenerating digit tip, the signaling pathways specific to the wound epidermis remain largely unknown. One established signaling function of the digit tip wound epithelium is the activation of the SDF-1α/CXCR4 pathway in mediating blastema formation (Lee et al., 2013). The chemokine SDF-1α is highly expressed in the wound epithelium and its receptor, CXCR4, is upregulated in the underlying mesenchyme (Figure 2A). Intriguingly, SDF-1α is not highly expressed in the wound epidermis of proximally amputated digits, but transplantation of SDF-1α-expressing cells to proximal amputations resulted in more regenerated bone, including an ectopic sesamoid-like bone (Lee et al., 2013) (Figure 3, Table 1). These experiments demonstrate the sufficiency of SDF-1α to increase regeneration and more broadly support the hypothesis that the wound epithelium is a unique, pro-regenerative tissue. However, both the regenerative P3 and non-regenerative P2 wound epithelia still require much investigation, particularly with regards to any differences in paracrine signaling.

### 4.2 Is it possible to stimulate regeneration in the mouse digit?

A long-term goal of the regenerative field is to improve and stimulate regeneration in tissues normally incapable of regenerating. While the distal digit innately regenerates under normal conditions, the proximal digit does not and can thus be used to test factors proposed to promote regeneration. This experimental set-up has been used successfully in other model organisms, such as pre- and post-metamorphic *xenopus* hindlimbs (Lin et al., 2013). However, even though the digit tip is an ideal model to “rescue” regeneration, no study to date has reported successful regeneration induced following a nonregenerative amputation to replace the entirety of the amputated digit (Figure 3, Table 1). A more nuanced perspective is that there are factors that have been established to help stimulate more regeneration than is typical in the fibrotic response. Techniques including conditional genetics, cell transplantation, agarose-bead implantation, and local or systemic injections have been utilized to assay certain factors that promote regeneration in the proximal P3 or P2 nonregenerative response. Such exogenous factors discussed in this review have ranged from established cellular signals such as BMP ligands and α-MSH to cells themselves, such as induced pluripotent stem cells or the entire nail organ. While none of these factors have yielded perfect pre-amputation digits, it is clear regeneration can be induced (Figure 3, Table 1). Thus, each experiment provides significant information into understanding the underlying signaling mechanisms between regeneration and fibrosis.

While the prospect of complete mammalian limb regeneration has not yet been fully realized, the field is making progress in understanding the requirements and limitations of the system. Much focus is on what makes the distal digit regenerative relative to the rest of the body, which is of course a major part of regenerative biology. However, while we discussed cellular heterogeneity, sources of progenitor cells, and important signaling pathways in digit tip regeneration, much of the same concepts are yet unknown in the context of the proximal fibrosing digit, which is arguably just as important to understand. While many questions remain, the field has made leaps and bounds to identify critical aspects of blastema-mediated regeneration. Ultimately, the collective findings from the mouse digit may be further extended for clinical and therapeutic applications in humans.
